# On Secale Cornutum, &c.

**Published:** 1840-01

**Authors:** Josiah Higgason

**Affiliations:** Somerville, Tenn.


### Art. III.—*Remarks on Secale Cornutum in Pseudocyesis Molaris.* By Dr. Josiah Higgason, of Somerville, Tenn.

In the latter part of December 1838, I was called to see Mrs. G., laboring under excessive uterine hemorrhage. So alarming was her exhaustion from loss of blood, that, together with other means, it was necessary to have recourse to the tampon. She was then directed to lie in bed, use light diet, keep her bowels open by rhubarb and magnesia, with occasionally a small dose of calomel, and when threatened with a return of hemorrhage, to use pills composed of opium, ipecacuanha and acetate of lead. For about a week she had frequent returns of flooding, generally light, but of sufficient severity to require the pill. She had also rigors, without the sensation of coldness, which I have often found to be indicative of the existence of some extraneous body within the uterus. In the intervals of hemorrhage, she had a discharge of a sanguineo-mucous character from the vagina, which had continued with greater or less profuseness for twelve months.

The patient indulged the belief that the solid parts of a fœtus were retained within the uterus, and that this was the source of all her suffering, which led to an examination. The uterus was found to be of the size which it attains about the third month of gestation, with the cervix elongated and the os tincæ rigid. The examination satisfied me that I had taken a wrong view of the case—I had regarded it as one of a disordered state of the menstrual function, and had prescribed for it as such. I was now at a great loss to determine its precise character. Did the uterus contain a dead fœtus? If so, it must have been retained for twelve months, since, during that period, her health had been too bad to admit of conception. About a year previously she was supposed to have aborted, and the retained substance, she believed, had existed within the uterus from that time. She recovered but imperfectly from the effects of the abortion, and continued to be pale and nervous, and to suffer with the discharge above referred to, which was occasionally so free, as to demand the use of astringent remedies, purgatives, &c. An alarming hemorrhage at the time specified, made it necessary to render her prompt and regular attention. She was found free from labor pains—the uterus had been quiescent for months—the cervix was firm, and the os uteri unyielding, but as the danger of the patient was imminent, it was determined, on consultation with Dr. Gray, a relative of the lady, to put her upon ten grain doses of ergot, every two or three hours, with a view to the production of light, but continued action in the uterus, thereby soliciting the relaxation of the parts. The remedy was commenced about 10 o'clock, A. M., and persevered in for four-and-thirty hours, when an organized, firm substance, about four inches long and two in diameter, was expelled. It was enveloped in a smooth, white, membranous coat, which had no appearance of having ever been attached to the uterus:—it was pear-shaped, corresponding to the cavity in which it had reposed, and in its structure spongy, or cellular, with tough, ligamentous bands, passing through it in different directions.

During the operation of the ergot, the following effects were observed with interest:—at first the hemorrhage was increased, but subsided when the tonic contractions of the uterus were established. After the first three doses, the patient complained of pain and uneasiness in the back, which gradually increased until the contractions became general, and occasionally severe. The remedy was then intermitted for a time, lest injury should result from too much pressure upon the still unyielding parts. Gradually, however, this rigidity was found to give way, until the cervix was obliterated, and the os tincæ relaxed, and the contents of the uterus expelled with but little pain. After having been established the pains never entirely ceased until the end was accomplished. The increase of hemorrhage upon the first administration of the remedy is always to be expected under similar circumstances. Repeatedly have I used it after abortion with a view, not only to the expulsion of portions of involucra, but mechanically to diminish the cavity of the uterus, thereby, in a manner, closing the mouths of the bleeding vessels, and always with this effect—first, an increase of the flooding, and then a diminution and suspension of it.

The above is the only case in which I ever employed ergot in a rigid state of the os uteri, and have to regret that I did not resort to it a year earlier, since the patient would have been rescued from many months of anguish and apprehension. The expulsion of the foreign body would, probably, have been effected with less difficulty, as the parts, it may be presumed, were in a more relaxed condition. In regard to the medicinal properties of this article, an unfortunate diversity of opinion prevails among medical men, being esteemed inert by some, and incapable, under any circumstances, of producing uterine contractions; by others, as efficient only at the full period of gestation; while not a few would discard it altogether on account of the uncertainty of its operation—small doses sometimes exciting violent contractions, and at others, under apparently a similar state of the case, large doses proving inefficient.

I have employed ergot in my practice for a dozen years, and have seldom been disappointed in its effects. I have used it in abortion, where that event was already unavoidable, to quicken the expulsion of the ovum, and have administered it immediately afterwards, to arrest hemorrhage by diminishing the uterine cavity. I have given it during labor to strengthen and accelerate the pains when the presentation was favorable, and the os uteri dilatable, where otherwise, doubtless, the forceps would have been required. In retention of the placenta, whether from adhesion or inertia, I have also administered the article, and uniformly with the desired effect. My mode is to give it in substance finely pulverized, in doses of from ten to thirty grains, at longer or shorter intervals, according to the urgency of the case and the effect produced, and my confidence in its specific action upon the womb is as great, as in the peculiar action of tartar emetic upon the stomach. Of the unfavorable occurrences said to result from its administration—hour-glass contractions, rupture of the uterus, &c.—I have seen none, but on the contrary in the only case of rupture of that organ which I ever witnessed, not a particle of the medicine had been employed.

The history of a single case of its employment in retained placenta will close these remarks. It is one of many of a similar character:

Mrs. ——— in the year 1837, had been delivered of an infant eighteen hours, without the supervention of labor pains, and in this inert condition of the uterus, the placenta was not expelled. I attempted to remove it by Velpeau's method, but without success. The hand was not introduced because my experience in the virtues of ergot taught me to expect that it might be extracted by an easier method. Thirty grains of the article were administered, and the dose was repeated in fifteen minutes. Uterine contractions were not long in coming on, and by one effort of the organ the placenta was expelled. The pains continued to be so violent that an opiate was thought necesary, showing that the last portion of the medicine ought not to have been given.

